# Behavioral, personality, and temperamental characteristics predict escalating alcohol and cannabis use and problems during the first-year of college

**DOI:** 10.21203/rs.3.rs-6157562/v1

**Published:** 2025-04-24

**Authors:** Chelsie E. Benca-Bachman, Dalora Najera, Colette N. Delawalla, Natalia Jaume-Feliciosi, John E. McGeary, Rohan H. C. Palmer

**Affiliations:** Emory University; Emory University; Emory University; Emory University; Providence VA Medical Center; Emory University

**Keywords:** Alcohol, Cannabis, College, Temperament, Transmissible Liability Index

## Abstract

Recent studies suggest that behavioral and temperament characteristics provide insight into individual differences in drug use behaviors, but few studies have described their prospective associations. We investigated how temperament and behavioral characteristics associate with drinking and cannabis use prior to and during the start of college. Participants were 252 college students (175 females, mean age = 18.56, *SD* = .38) from the MAPme Project. Behavioral and temperamental characteristics were assessed using the Transmissible Liability Index [TLI]) in orer to predict alcohol and cannabis use and problems assessed using the Alcohol, Smoking and Substance Involvementprior scale. Linear and logistic regression was used to assess associations at and across each wave of assessment. Roughly 61% of students used alcohol and 27% used cannabis in the three months prior to starting college. Higher TLI scores were associated with earlier age of alcohol and tobacco initiation, greater odds of drinking alcohol (3.41 [95% CI = 1.90,6.12]) and smoking cannabis (3.79 [7.86,7.73]) during the first semester, and more more severe alcohol problems at college (β = 0.31 [0.09,0.56]). The findings illuminate the sensitivity and utility of temperament and behavioral characteristics in forecasting risky behaviors, highlighting an opportunity to better structure campus drug prevention.

## Introduction

Substance use by college students is a complex phenomenon that occurs at the nexus of increased freedom, decreased parental oversight, increases in risk-taking propensity, the establishment of new social networks, social norms around drinking, and the availability of substances [1, 2]. These unique collection of factors occurs at a time where adult responsibilities are relatively low allowing individuals to use different substances with more freedom and fewer consequences compared to in adolescence or middle adulthood [3]. Substance use remains prevalent despite the existence of policies meant to curtail risky drug and alcohol use across university campuses [4].

### Epidemiology and consequences of college alcohol and marijuana use

Previous studies demonstrate increases in alcohol consumption—particularly binge-drinking behaviors during the first year of college—are associated with more alcohol-related consequences [5]. White, Kraus [6] found that close to 20% of male college students and 10% of female college students consumed twice the binge threshold (five and four drinks, respectively) in the two weeks preceding their study. Almost one-third of students entering college have reported using marijuana and 8.5% of those who had not, started during their first year [7]. While attending college, one in five full-time students go on to meet criteria for DSM-IV alcohol use disorder [8, 9] and one in ten are expected to meet criteria for cannabis use disorder [8]. Importantly, most who engage in risky substance use during college tend to “mature out” of problematic use as adult responsibilities increase [3]. However, identifying college students who use substances and preventing risky use continues to be an academic imperative for at least two important reasons: 1) preventing adverse consequences during this time of increased use and 2) intervening in the subset of inidividuals at heightened risk of failing to “mature out” and develop long term substance use problems.

In addition to the many health and safety consequences associated with excessive substance use (e.g., motor vehicle crashes, violence, miscarriages or negative birth outcomes, and engaging in risky behaviors [10]) there exists academic consequences associated with some substances. Suerken, Reboussin [7] found that compared to non-users, students who used cannabis frequently, including those whose use decreased over time, were more likely to drop out of college and plan to delay graduation. Likewise, cannabis use has been associated with lower GPA, skipping class, and extended college completion timelines [7, 11]. By contrast, findings on the academic outcomes related to alcohol use in college suggest academic performance and alcohol involvement are less clearly delinated. Some research has found alcohol use to have only a modest negative association with academic outcomes, that disappear when covariates are included in modeling [12–14], while other research suggests that heavy episodic drinking is associated with negative academic outcomes by way of how important one perceives their grades to be [15]. Taken together, although experimentation with substance use is common in college students given the negative associations with health, safety, and academic performance it is important to work toward delaying or preventing alcohol and cannabis use in this population [11, 16, 17].

### Assessing substance use disorder risk

Researchers and university administrations have long targeted campus drug prevention by leveraging research-driven frameworks, such as the NIAAA’s CollegeAIM [18] initiative that helps universities identify individual and environmental level evidenced-based intervention strategies to reduce student alcohol use. While college student self-reported substance use patterns are mostly accurate, in a research context [19], other studies suggest this is not the case when reporting use to non-research related university entities [20]. This is unsurprising given many universities have punitive policies in place regarding substance use, providing students little incentive to be honest about their use when asked, even in an anonymous survey form (e.g., Dry Campus policies). Patterns of underreporting are particularly problematic when considering that university efforts to implement interventions rely on these self reports. One potential solution is to use a measure that is not face valid for substance problems but can identify individuals who are currently using and those who are likely to go on to use substances.

The Transmissible Liability Index (TLI) has been suggested as an assessment tool for measuring the common, or shared, propensity for substance use disorders for the purposes of etiological research and the development of individualized interventions [21]. The underlying rationale behind the TLI stems from the theory that shared and non-shared genetic and environmental factors across multiple behaviors and traits contribute to individual differences in substance use disorder (SUD) liability among individuals in families and the broader population [22, 23]. Previous research on the TLI has demonstrated its ability to (1) independently capture risk for SUDs beyond family history, (2) account for the aggregate effects of other putative risk factors for SUD beyond substance-specific risk, and (3) reflects the shared vulnerability across the major contributing behavioral and personality factors that contribute to SUD liability [24]. For instance, the set of TLI items includes a respondents’ history of internalizing and externalizing problems that are associated with risk for developing substance use disorder. These items characterize individual behavioral and personality constructs (e.g., antisocial traits, impulsivity, and mood) that are not specific to use of substances and may be present prior to first use. In short, the covariance structure across the TLI items can be modeled and employed as a multivariate liability index. Prior applications of the TLI in college-aged populations have been limited. Arria, Vincent [25] conducted a validation study of the TLI in a sample of students drawn from the College Life Study. This study has shown that TLI scores are significantly higher not only among students with substance use disorders at baseline, but also among students who go on to develop alcohol and cannabis problems in the following two to four years. However, the authors did find that specificity and sensitivity were lacking. Brown et al. 2020 have also shown that higher scores on the TLI are associated with an earlier age of onset of drinking and greater family history of drug and alcohol use and internalizing problems.

### Study Goals

The current study examined how personality and behavioral characteristics, captured by the TLI, predict alcohol and cannabis initiation, use, and related problems during the first semester of college. We investigated the TLI’s ability to predict substance use and related problems during the transition into college. Specifically, data were collected by retrospective self-report sometime during the first six weeks of students’ first semester in college (i.e., baseline/Wave 1 [W1]) and midway through their first semester (Wave 2 [W2]), which was, on average 2.5 months later (minimum = 1.5, maximum = 3.5). We hypothesized that higher TLI scores would: 1) be associated with greater use of alcohol and cannabis at both waves, 2) be associated with earlier age of initiation of alcohol and cannabis use, and 3) demonstrate predicitive utility over and above self-reported age of first use and prior substance problems. Importantly, this work adds to the literature by taking a dimensional approach to quantifying problems associated with risky use and by only including information in the analyses that universities would be privy to (e.g., information about parental use or prior problems is not included). In taking this approach the TLI’s ability to predict problems through the transition into college in the same way universities might use the information to identify students at risk, was tested.

## Materials and Methods

### Sample

Participants were first year students recruited from multiple campuses at Emory University to partipate in a pilot project entitled, Multidimensional Assessment of Addiction Problems in Me Project’s (MAPme Project; baseline N = 304; subset with data across waves N = 252). Individuals were recruited during the first week of the academic semester in Fall 2018. All participants were required to provide consent to participate in the study and the associated MAPme Data Repository. Students participating in MAPme were required be at least 18 years of age and in their first year of college at the time of consent.

Data were gathered using self-report assessments across six weeks at baseline (μ_completion week_ = 3.11, (Standard Deviation [SD] = 1.77) and again across four weeks near the end of the first semester (W2). Study data were collected and managed using REDCap electronic data capture tools hosted at Emory University [26]. Recruitment flyers and events were directed at the entirety of the incoming freshman class that was 38% White, 24% Asian, 11% Hispanic, 7% African American, and 4% multi- racial/ethnic groups; 16% self-identified as non-US citizens rather than a racial category. Each student who provided consent and joined the study received a $15 gift card after completing the web assessments and an additional $5 if they provided a saliva sample. The current study focused on 252 students (70% female; μ_age_ = 18.56 [SD = 0.38]) who responded to invitations to participate at the second wave of data collection (86% response rate).

### Human Ethics and Consent to Participate

The Emory University IRB approved the study (IRB #: 00096137), which was conducted in accordance with Helsinki Declaration as revised in 2013 (Clinical trial number: not applicable). As part of the consent process, participants consented to the the publication of manuscripts, using their responses, in scientific journals.

### Assessments

#### Transmissible Liability Index

Students were administered the young-adult version of the Transmission Liability Index (TLI) adapted by Ridenour, Kirisci [24] at baseline. Similar to prior work in college populations by Arria, Vincent [25], the multifactorial index of SUD liability was derived using items that were drawn from constructs associated with SUD risk in previous research (e.g., conduct/antisocial personality disorder, illegal or aggressive behavior, mood, anxiety and personality traits). Higher scores on this unidimensional construct have been shown to indicate greater risk for generalized substance use problems and vice versa [24], as well as family history of internalizing psychopathology and pre-collegiate alcohol use [27]. TLI scores were obtained using a confirmatory factor model based on participants’ answers to 24 “yes/no” questions (see Table 1 for item endorsement and factor loadings using items with sufficient endorsement rates; five items were excluded due to low endorsement (i.e., number of individuals endorsing an item was less than eight)). This approach allowed for the unique contribution of different items to be included in one’s liability score, thus providing a more accurate understanding of “liability” as prior research has indicated that each item does not contribute equally to propensity for risk consumption [28]. The model provided a satisfactory fit to the set of items (Root Mean Square Error of Approximation (RMSEA) = 0.06, [90% CI = 0.05, 0.07].[29] Other factor structures were not explored given that model fit was adequate and theoretical considerations of a generalized liability toward substance use was more relevant to the current study than potential sub-factor models. Analyses employed scores on the latent variable after dropping item 13 (due to low endorsement), which did not change the model fit (RMSEA = 0.06 [0.05,0.07]); TLI scores were similar across sexes (β_male_vs_female_ = −0.11 [95% CI = −0.23, 0.01]); see supplemental Table S1 for inter-item correlations. Analyses employed a standardized factor score (mean = 0, standard deviation = 1).

#### Alcohol and Cannabis Use and Problem Severity

Alcohol and cannabis involvement were assessed at both waves using relevant items from the World Health Organization’s Alcohol, Smoking, and Substance Involvement Screening Test (ASSIST, V3.0) substance involvement scoring criterion [30]. The ASSIST is a self-report assessment that asks respondents about lifetime use of nine classes of substances and frequency of experiences related to substance-specific use in the past three months. At baseline, lifetime use of alcohol or cannabis (1 + times) was based on a single item on the ASSIST scale. At Wave 2, use was defined as “any use (1 + times) in the past three months” OR “since the baseline assessment”. In order to ascertain age of initiation, an item asking age at the time of first use was added following first endorsement of lifetime use, whether first endorsement was at Wave 1 or Wave 2. Indices of problematic substance use were derived using (1) frequency of use in the past three months (W1) or since the prior assessment (W2), and any of the following symptoms during the same time period: (2) cravings; (3) ‘failure to fulfill obligations’, (4) ‘health, social, legal, or financial problems’, (5) ‘expression of concern from someone’, and (6) ‘failure to cut down or stop using’. Responses were on a Likert scale from 0 (“Never” or “No”) to 6 (“Daily or Almost Daily” or “Yes, In the past 3 months/since the last assessment”). As such, substance-specific problem indices can range from 0 to 36 before log transformation (using log-transformed scores (Ln([AUP/CUP symptom count] + 1))) with higher scores indicating a higher severity of alcohol and cannabis use problems (referred to as AUP and CUP, respectively). Non-users were coded as 0, and were *not* incorporated into the category of “missing”.

### Statistical Approach

Sample characteristics were examined using R for Statistical Computing [version 3.3.3] [31]. Variables were examined for normality using univariate procedures. Binomial logistic regression in MPlus (version 8) was used to examine the association between TLI and alcohol and cannabis use (dichotomous “yes”/”no”) and age of initiation before and after accounting for the effects of covariates (i.e., “covariates only” and “covariates + TLI”, respectively) at both W1 and W2. Linear regression was used to model the prediction of TLI scores on alcohol or cannabis age of initiation and problems using a similar approach. Alcohol/Cannabis problems at each wave was first examined using only the covariates (age, sex, campus, completion week, and age of first use of each substance) as predictors (i.e., “covariates only”). The model was then expanded to include TLI (i.e., “Covariates + TLI”). Lastly, we examined the robustness of the TLI effect, by further expanding the model to include age of first use of the substance (i.e., “covariates + TLI + prior drug info”). When predicting W2 substance problems (AUP/CUP) we repeated the first two models and accounted for age of initiation and prior history of problems observed at Wave 1. R-squared estimates, odds-ratios, and standardized regression coefficients are reported.

## Results

### Description of study variables

Table 2 provides a description of alcohol and cannabis use and ASSIST total scores at both waves of assessment. Sixty one percent of students had tried alcohol and 27% had tried cannabis at baseline. By comparison, these levels were lower at Wave 2, with 53% reporting having used alcohol one or more times since the baseline assessment and 19% reported having used cannabis. The average reported age of alcohol initiation was 16.41 (SD = 2.08) years and cannabis initiation was 17.05 (SD = 1.33) years. At baseline AUP scores ranged from 2 to 26 (Mean [μ] = 6.51, SD = 4.47) while CUP ranged from 1 to 35 (μ = 7.6, SD = 6.35). At Wave 2, AUP and CUP scores ranged from 1 to 35 (μ = 7.02, SD = 5.07) and 2 to 29 (μ = 8.85, SD = 6.10), respectively.

### Cross-sectional associations between TLI and substance involvement

TLI predicted individual differences in likelihood of reporting alcohol and cannabis use prior to and during college (see Table 3). Higher scores on the TLI were associated with a greater likelihood of alcohol and cannabis use at both waves (e.g., Wave 2: Alcohol Odds Ratio = 3.09, [95% CI = 1.77, 5.42]; β = 0.29 [0.16, 0.43]); Cannabis OR = 3.79, [1.86, 7.73]; β = 0.33 [0.17, 0.49]). Higher TLI was also associated with earlier age of use initiation of alcohol and cannabis (Alcohol: β = −0.23 [−0.38, −0.09]; Cannabis: β = −0.30 [−0.51, −0.08])).

Table 4 shows the results of the multiple regression models in which we examined the association between TLI and severity of substance problems upon entering college. Higher TLI scores were associated with greater cannabis problems and was not confounded with the effects of earlier age of onset of use. Notably, the TLI effect was not confounded with self-reported age of initiation of alcohol use and demonstrated a robust effect at Wave 1. However, while the effects in the model predicting CUP were trending in a similar pattern, they did not meet significance criteria in this sample.

### Prospective associations between TLI and alcohol and cannabis problems

At Wave 2, TLI scores were the most robust predictor of alcohol problems. As hypothesized, TLI scores explained variance in AUP over and above self-reported age of initiation and problems reported at Wave 1 (β = 0.31, [0.085, 0.56]). TLI was not robustly associated with cannabis problems during the first semester of college; while there appeared to be a trend-level effect in the model including ‘TLI and covariates’, the observed effect size was reduced and uncertainty of the prediction greater when accounting for ‘age of initiation of cannabis’ and ‘prior history of cannabis problems’. Only prior problematic use of cannabis robustly predicted cannabis problems during the first semester at college.

## Discussion

### Summary and Explanation of Results

To date, studies employing the composite behavioral, personality, and termperamental characteristics, such as the TLI in college populations have been limited. The current study expands this body of work by evaluating the robustness of the TLI in predicting alcohol or cannabis use-related problems over a significantly shorter period than has previously been attempted. We took a fine-grained approach to testing the utility of temparement and behavioral characteristics in identifying those who may be at risk for escalation of substance use and related problems during the transition into a college setting. In support of our hypotheses, we found that higher scores on the TLI were associated with alcohol and cannabis use and lower self-reported age of initiation of both substances. Further, our results suggested the TLI provided predictive utility for those exhibiting alcohol problems over and above explicit self-reported use and problems measures. Contrary to our expectations, the best predictor off future cannabis use problems was past problems, not the TLI.

In both research and university resources like CollegeAIM [18], heavy emphasis is placed on identifying and engaging at risk individuals as early as possible as they transition into the college environment [32] —a task which requires efficient assessment of student substance use. However, there is an important distinction between collecting self-reported substance use information at a research level (i.e., participating in research studies) versus at the university administration level (i.e., in the form of questionnaires at orientation). Specifically, that while students tend to report alcohol use accurately in research settings [19], the same may not be said of reporting use to university administration where punitive policies may be in place. The present work addresses this issue by providing support for the employment of a measure that can capture risk for alcohol problems without explicitly asking students to report illegal or excessive alcohol use.

Results for cannabis use, although not in line with our hypotheses, are in line with work suggesting the best predictor of future problems are past problems [18]. Although the current study was not well-powered to detect TLI’s effect on CUP upon entering college, the trending effect suggests that the TLI may capture some risk liability—just not more than is accounted for by assessing past problems. Our results of cannabis use during this transitionary period highlight a number of important considerations. First, self-reported cannabis use *decreased* between Waves 1 and 2. College, is a heterogeneous pool of individuals from different states and countries which vary in their enforcement, acceptance, and attitudes towards recreational and medicinal use of cannabis [33]. Given the illegality of cannabis in the state where this study was conducted, such use would have likely required students to have connected with a source—this relationship building in a new environment can take time. Second, it is also possible that the lack of a TLI effect on CUP may be a result of individual differences in expectations and attitudes toward cannabis smoking that was not captured in the current study. By comparison, drinking is more commonly perceived to have positive effects among college students, it is also more easily accessible.[34] As such, differences in exposure and the persistence of the TLI effect over time could be moderated by expectations, though further research is needed to confirm this.

### Limitations

A notable limitation of our study is that we lacked sufficient data to demonstrate that these effects generalize to other types of academic institutions, such as public and two-year colleges and universities. Yet, our findings are generally consistent with the earlier report by Arria et al [25], which showed a positive association between TLI and substance use at a public institution. Second, we note the current study did not examine the generalizability of these pattern of effects to different racial/ethnic groups and sexes due to lack of power, nor did the current study investigate possible unique effects of using both cannabis and alcohol that have been suggested by previous studies [16]. Third, the TLI does include face valid items about the individual’s substance use which may be prone to underreporting. However, due to the breadth of content and the scoring approach used in this study, propensity for risk can still be captured even if the individual underreports those items.

## Conclusions & Future Directions

Despite these limitations, the present study advances our understanding of the utility of a brief, self-report instrument in identifying the potential for developing problems related to alcohol use over a period of weeks among a longitudinal sample of students in the first year of college, regardless of prior experience. Although the TLI did not provide predictive utility of problems with cannabis use over and above reporting past problems, the prospective project from which the current study was derived is ongoing. It is possible to examine whether the TLI will predict cannabis-related problems at later waves. Many individuals who initiate will no longer use substances after a short period of experimentation, while some adolescents and young adults will either maintain their levels of use without problems, or progress to patterns of substance abuse characterized by both psychological and physiological dependence, or substance use disorder [35]. Once problems related to substance use escalate to the point of meeting the clinical criteria for a substance use disorder, few young adults who require treatment actually receive it. According to the NSDUH (2018), among the 5.2 million (15.3%) people between the ages of 18 and 25 who meet criteria for a substance use disorder (SUD) only 6.3 percent receive treatment. Additionally, most young adults who meet criteria for a substance use disorder (SUD) are not receptive to formal treatment. The number of young adults who met criteria for a SUD and did not receive treatment who felt they needed it was only 3.4% (NSDUH). Therefore, young adults are not likely to actively seek out treatment despite the presence of detrimental problems. Further, once people feel that they need treatment, perceived barriers to obtaining treatment include not being ready to stop, not being able to afford the cost of healthcare, not knowing where to get treatment, that others will have a negative opinion of them as a result, and that it would negatively impact their careers (NSDUH). As such, early identification of risk and prevention is the point along the continuum of care where the TLI can be useful for colleges and universities aiming to address substance use among its students.

Overall, these findings suggest that retrospective self-report characteristics of students prior to entering college can be used to index the risk for alcohol problems and cannabis use. Taken together, this indicates that colleges can aid in the reduction of risking alcohol use of their students by using an efficient method of quantifying and identifying individuals at increased risk so that protective interventions and tools can be developed, staffed, and implemented in a more strategic manner.When choosing and planning for prevention efforts, colleges may focus on the general population model (universal), target high risk groups (selective), or target those students showing early danger signs or behaviors (indicated) [36]. Whereas universal interventions may use resources on the significant portion of students who will not develop problems, prior to the development of problems that would usually signal the need for an indicated intervention, the TLI can assist with identifying students who are most likely to benefit from selective interventions whose risk may not otherwise be identified. The TLI is cost-effective, quick, and precise. The assessment does not ask students to disclose their involvement with alcohol or cannabis, which they may not be inclined to do for fear of disciplinary action. Rather than relying on traditional methods of identifying at-risk students, such as help-seeking, declining grades, and absenteeism, the TLI has the potential to be used to screen all students so that prevention resources can be allocated efficiently and employed before students experience these disruptions in academic progress.

## Figures and Tables

**Table 1:** TLIa indicators and standardized factor loadings

	Prevalence	Factor Loadings		
Item Description		β	SE	P
1. In your entire life, have you ever had a time, lasting 2 weeks, when you didn’t care about the things you usually care about, or you didn’t enjoy what you usually enjoy?	0.48	0.738	0.046	<0.001
2. Have you ever had a time that lasted for at least 2 years when your mood was low, sad, or depressed most of the day, more than half of the time?	0.27	0.651	0.059	<0.001
3. Have you ever had a strong fear or avoidance of being in a crowd or standing in a line?	0.32	0.485	0.066	<0.001
4. Most of the time throughout your life, regardless of the situation or whom you were with, have you avoided jobs or tasks that dealt with a lot of people?	0.26	0.515	0.068	<0.001
5. Do you often have to keep an eye out to keep people from using you, hurting you, or lying to you?	0.35	0.741	0.046	<0.001
6. Do you spend a lot of time wondering if you can trust your friends or the people you work with?	0.30	0.712	0.052	<0.001
7. Do you find that it is best not to let other people know much about you because they will use it against you?	0.30	0.695	0.055	<0.001
8. Do you often get angry or lash out when someone criticizes you in some way?	0.18	0.482	0.078	<0.001
9. Did you ever have a time in your life when you lied a lot or any time you lied to keep from being hurt?	0.35	0.664	0.048	<0.001
10. Did you ever have a time in your life when you lied a lot, NOT counting any times you lied to keep from being hurt?	0.19	0.650	0.06	<0.001
11. Did you ever get more than 3 traffic tickets for reckless or careless driving, speeding, or causing an accident?	0	N/A^b^	N/A^b^	N/A^b^
12. Did you ever run away from home overnight at least twice when you were living at home, or run away and stay away for a longer time?	0.04	0.688	0.104	<0.001
13. Did you ever destroy, break, or vandalize someone else's property like their car, home, or other personal belongings?	0.04	0.131	0.107	0.221
14. Did you ever fail to pay off your debts- like moving to avoid paying rent, not making payments on a loan or mortgage, failing to make alimony or child support payments or filing for bankruptcy?	0.01	N/A^b^	N/A^b^	N/A^b^
15. Did you ever steal anything from someone or someplace when no one was around?	0.19	0.573	0.069	<0.001
16. Did you ever shoplift?	0.12	0.599	0.077	<0.001
17. Did you ever rob or mug someone or snatch a purse?	0	N/A^b^	N/A^b^	N/A^b^
18. Did you ever get into a lot of fights that you started?	0.02	0.922	0.068	<0.001
19. Did you ever get into a fight that came to swapping blows with someone like a husband, wife, girlfriend, or boyfriend?	<0.01	N/A^b^	N/A^b^	N/A^b^
20. Did you ever hit someone so hard that you injured them or they had to see a doctor?	0.03	0.400	0.15	0.007
21. Did you ever physically hurt another person in any other way on purpose?	0.11	0.418	0.096	<0.001
22. Did you ever harass, threaten, or blackmail someone?	0.03	0.879	0.077	<0.001
23. Did you ever make money illegally like selling stolen property, or selling drugs?	0.02	0.690	0.14	<0.001
24. Did you ever use a weapon like a stick, knife, or gun in a fight?	0	N/A^b^	N/A^b^	N/A^b^

Table of factor loadings on latent trait using items with sufficient endorsement rates for analysis.

Notations:

a Transmissible Liability Index,

b N/A - excluded from model due to low level of endorsement. See supplemental Table S1 for inter-item correlations where numbers 1 thru 24 correspond to items TLI_1 thru TLI_24.

**Table 2.** Descriptive statistics of study variables at each wave of assessment

N = 304	n		%	mean	SD	median	min	max	range
Age	252			18.56	0.38	15.56	18.01	20.39	2.38
Sex	251		70% Female						
Completion week	252			3.10	1.77	2.95	1	8	7
Campus	252		67% Urban						
TLI	252			0.04	0.49	0.04	−0.65	1.41	2.06
Wave 1									
Ever used Alcohol	153		61%						
Ever used Cannabis	68		27%						
Age of first Alcohol Use	150			16.41	2.08	17	8	19	11
Age of first Cannabis use	66			17.05	1.33	17	13	19	6
Log transformed AUP	139			1.69	0.67	1.61	0.69	3.26	2.56
Log transformed CUP	55			1.77	0.70	1.76	0.69	3.47	2.77
Untransformed AUP	139			6.51	4.47	5	2	26	24
Untransformed CUP	66			7.60	6.35	6	1	35	34
N = 252			Wave 2						
Used Alcohol since W1	132		53%						
Used Cannabis since W1	48		19%						
First Alcohol use since W1	8		3%						
First Cannabis use since W1	6	2	2%						
Log transformed AUP	131			1.76	0.61	1.79	0	3.56	3.56
Log transformed CUP	47			1.98	0.66	2.08	0.69	3.37	2.67
Untransformed AUP	132			7.02	5.07	6	1	35	34
Untransformed CUP	48			8.85	6.10	8	2	29	27

Note: AUP = Alcohol use related problems, CUP = Cannabis use related problems, min = minimum, max = maximum.

**Table 3.** Regression results for models examining the association between TLI, Age of initiation, and alcohol & cannabis use

Models	Covariates only		Covariates + TLI	
*Age of initiation of alcohol use*	b [95% CI]	β [95% CI]	b [95% CI]	β [95% CI]
Sex (male)	0.45 [−0.25,1.15]	0.10 [−0.06,0.24]	0.30 [−0.40,0.98]	0.06 [−0.09,0.22]
Age	−0.31 [−1.13,0.52]	−0.06 [−0.22,0.09]	−0.33 [−1.13,0.47]	−0.06 [−0.21,0.09]
Week	−0.03 [−0.22,0.15]	−0.03 [−0.17,0.13]	−0.06 [−0.24,0.12]	−0.05 [−0.20,0.10]
Campus (urban)	0.52 [−0.17,1.09]	0.12 [−0.05,0.25]	0.53 [−0.13,1.20]	0.12 [−0.03,0.27]
TLI^b^	N/A	N/A	−0.98 [−1.61,−0.34]^b^	−0.23 [−0.38,−0.09]^b^
*model r-squared [95% CI]*	0.03 [−0.02, 0.06]		0.08 [<0.01,0.06]	

*Age of initiation of cannabis use*	b [95% CI]	β [95% CI]	b [95% CI]	β [95% CI]
Sex (male)	0.17 [−0.47,0.80]	0.06 [−0.17,0.29]	0.03 [−0.59,0.65]	0.01 [−0.21,0.23]
Age	0.55 [−0.29,1.40]	0.15 [−0.08,0.38]	0.68 [−0.16,1.46]	0.19 [−0.04,0.39]
Week	−0.10 [−0.29,0.09]	−0.13 [−0.35,0.10]	−0.15 [−0.33,0.03]	−0.19 [−0.40,0.04]
Campus (urban)	−0.16 [−0.77,0.50]	−0.06 [−0.27,0.17]	−0.14 [−0.72,0.46]	−0.05 [−0.27,0.17]
TLI^b^	N/A	N/A	−0.75 [−0.19,−0.03]^a^	−0.30 [−0.53,−0.10]^a^
*model r-squared [95% CI]*		0.04 [−0.05,0.12]		0.12 [<−0.01, 0.36]

*Alcohol use at Wave 1*	Odds-ratio [95% CI]	β [95% CI]	Odds-ratio [95% CI]	β [95% CI]
Sex (male)	0.70 [0.40,1.22]	−0.109 [−0.23,0.05]	0.79 [0.44,1.42]	−0.06 [−0.20,0.08]
*Age*	1.35 [0.66,2.76]	0.06 [−0.09,0.21]	1.34 [0.65,2.76]	0.06 [−0.08,0.20]
Week	1.15 [0.98,1.33]	0.13 [−0.01,0.27]	1.19 [1.02,1.39]^a^	0.16 [0.02,0.30]^a^
Campus (urban)	0.72 [0.42,1.23]	−0.09 [−0.23,0.05]	0.66 [0.38, 1.16]	−0.10 [−0.24,0.04]
TLI^b^	N/A	N/A	3.41 [1.90,6.12]^b^	0.30 [0.16,0.45]^b^
*model r-squared [95% CI]*		0.04 [−0.02,0.08]		0.13 [0.03,0.21]^a^

*Alcohol use at Wave 2*	Odds-ratio [95% CI]	β [95% CI]	Odds-ratio [95% CI]	β [95% CI]
Sex (male)	0.90 [0.52,1.56]	−0.03 [−0.16,0.11]	1.03 [0.58,1.83]	0.01 [−0.13,0.14]
Age	0.90 [0.46,1.75]	−0.02 [−0.16, 0.12]	0.89 [0.45,1.76]	−0.02 [−0.16,0.11]
Week	1.05 [0.91,1.22]	0.05 [−0.09,0.19]	1.09 [0.94,1.26]	0.08 [−0.06,0.22]
Campus (urban)	0.77 [0.45,1.30]	−0.07 [−0.21,0.07]	0.71 [0.41,1.23]	−0.08 [−0.22,0.05]
TLI^b^	N/A	N/A	3.09 [1.77,5.41]^b^	0.29 [0.16,0.43]
*model r-squared [95% CI]*		0.01 [−0.02,0.03]		0.09 [0.01, 0.17]^a^

*Cannabis use at Wave 1*	Odds-ratio [95% CI]	β [95% CI]	Odds-ratio [95% CI]	β [95% CI]
Sex (male)	1.02 [0.55,2.30]	0.00 [−0.15,0.16]	1.16 [0.61,2.20]	0.04 [−0.12,0.19]
Age	1.25 [0.60,3.28]	0.05 [−0.11,0.20]	1.26 [0.59,2.69]	0.05 [−0.10,0.24]
Week	1.18 [1.00,1.45]	0.15 [−0.01,0.20]	1.22 [1.04,1.44]^a^	0.18 [0.04,0.33]^a^
Campus (urban)	1.03 [0.57,1.90]	0.01 [−0.15,0.16]	0.97 [0.53,1.78]	−0.01 [−0.16,0.14]
TLI^b^	N/A	N/A	2.97 [1.60,5.52]^b^	0.28 [0.13,0.40]^b^
*model r-squared [95% CI]*		0.028 [−0.02,0.07]		0.11 [0.01,0.20]^b^
*Cannabis use at Wave 2*	Odds-ratio [95% CI]	β [95% CI]	Odds-ratio [95% CI]	β [95% CI]
Sex (male)	1.08 [0.54,2.16]	0.02 [−0.15,0.19]	1.29 [0.63,2.66]	0.06 [−0.11,0.23]
Age	0.89 [0.38,2.09]	−0.02 [−0.20,0.15]	0.90 [0.37,2.21]	−0.02 [−0.19,0.15]
Week	1.18 [0.99,1.41]	0.16 [<−0.01,0.32]	1.25 [1.04,1.50]^a^	0.20 [0.04,0.36]^a^
Campus (urban)	0.65 [0.32,1.31]	−0.11 [−0.29,0.07]	0.58 [0.28,1.21]	−0.13 [−0.31,0.04]
TLI^b^	N/A	N/A	3.79 [1.86,7.73]^b^	0.33 [0.17,0.49]^b^
*model r-squared [95% CI]*		0.04 [−0.02,0.10]		0.15 [0.03,0.27]^a^

Notations: CI = Confidence Interval, TLI = Transmissible Liability Index

a p-value < 0.05

b p-value < 0.001

N/A – not applicable

**Table 4.** Standardized regression (β [95% Confidence Interval]) results for models of AUP^a^ and CUP^b^ at each wave

	Wave 1			Wave 2		
*Predictors of AUP*	Covariates only	Covariates + TLI	Covariates + TLI + Prior drug info	Covariates only	Covariates + TLI	Covariates + TLI + Prior drug info
Sex (male)	0.12 [−0.05,0.28]	**0.14 [−0.03,0.319]** ^a^	0.03 [−0.20,0.25]	0.06 [−0.12,0.23]	0.08 [−0.09,0.25]	0.15 [−0.09,0.38]
Age	−0.04 [−0.20,0.13]	−0.04 [−0.20,0.13]	−0.12 [−0.34,0.10]	0.03 [−0.15,0.20]	0.03 [−0.14,0.20]	0.06 [−0.17,0.29]
Week	**0.07 [−0.10,0.23]** ^a^	**0.08 [−0.09,0.24]** ^a^	0.09 [−0.13,0.30]	0.02 [−0.15,0.20]	0.04 [−0.13,0.21]	−0.03 [−0.19,0.25]
Campus (urban)	−0.02 [−0.15,0.18]	−0.001 [−0.17,0.17]	−0.03 [−0.25,0.19]	0.10 [−0.08,0.27]	0.09 [−0.08,0.26]	0.05 [−0.18,0.28]
TLI^c^	N/A	0.10 [−0.07,0.27]	0.06 [−0.18,0.29]	N/A	0.20 [0.04,0.37]^a^	**0.31 [0.09,0.56]** ^a^
Age of Initiation	N/A	N/A	−0.08 [−0.14,0.30]	N/A	N/A	−0.08 [−0.31,0.16]
Wave 1 AUP	N/A	N/A	N/A	N/A	N/A	−0.04 [−0.27,0.18]

*model r-squared [95% CI]*	0.08 [−0.01,0.17]	0.03 [0.04,0.26]	0.03 [0.05,0.29]	0.01 [−0.03,0.11]	0.05 [0.01,0.22]	0.12 [0.37,0.63]

*Predictors of CUP*						
Sex (male)	0.18 [−0.07,0.51]	0.22 [−0.04,0.72]	0.21 [−0.02,0.45]	0.05 [−0.23,0.34]	0.09 [−0.19,0.37]	0.02 [−0.26,0.29]
Age	−0.13 [−0.38,0.21]	−0.15 [−0.79,0.19]	−0.06 [−0.31,0.20]	0.15 [−0.13,0.43]	0.13 [−0.14,0.40]	0.11 [−0.16,0.39]
Week	0.05 [−0.21,0.39]	0.12 [−0.06,0.17]	0.07 [−0.18,0.33]	0.15 [−0.12,0.42]	0.22 [−0.05,0.49]	−0.07 [−0.37,0.22]
Campus (urban)	0.14 [−0.12,0.40]	0.15 [−0.15,0.58]	0.13 [−0.11,0.37]	0.22 [−0.05,0.49]	0.19 [−0.08,0.45]	−0.06 [−0.33,0.24]
TLI^c^	N/A	**0.30 [0.06,0.79]** ^a^	0.20 [−0.06,0.46]	N/A	0.25 [−0.02,0.53]	0.05 [−0.25,0.34]
Age of Initiation	N/A	N/A	−**0.28 [−0.54,−0.02]** ^b^	N/A	N/A	−0.17 [−0.50,0.14]
Wave 1 CUP	N/A	N/A	N/A	N/A	N/A	**0.59 [0.32,0.85]** ^a^

*model r-squared [95% CI]*	0.07 [−0.06,0.20]	0.15 [−0.01,0.35]	0.21 [0.03,0.42]^a^	0.10 [0.06,0.26]	0.15 [−0.03,0.36]	0.43 [0.17,0.68]^b^

Notations: AUP = Alcohol-use related problems, CUP = Cannabis-use related problems, TLI = Transmissible Liability Index, CI = Confidence Interval

a p-value < 0.05

b p-value < 0.001

N/A – not applicable

## Data Availability

Data are available upon request to the senior author as they are already a part of a repository.

## References

[1] Stone A.L., , Review of risk and protective factors of substance use and problem use in emerging adulthood. Addict Behav, 2012. 37(7): p. 747–75. 22445418 10.1016/j.addbeh.2012.02.014

[2] Josef A.K., , Stability and change in risk-taking propensity across the adult life span. J Pers Soc Psychol, 2016. 111(3): p. 430–50. 26820061 10.1037/pspp0000090 PMC5470073

[3] O’Malley P.M., Maturing out of problematic alcohol use. Alcohol Research & Health, 2004. 28: p. 202–204.

[4] Toomey T.L., , Enforcing alcohol policies on college campuses: reports from college enforcement officials. J Drug Educ, 2011. 41 (3): p. 327–44. 22125925 10.2190/DE.41.3.f

[5] Krieger H., , The Epidemiology of Binge Drinking Among College-Age Individuals in the United States. Alcohol Res, 2018. 39(1): p. 23–30. 30557145 10.35946/arcr.v39.1.05 PMC6104967

[6] White A.M., Kraus C.L., and Swartzwelder H., Many college freshmen drink at levels far beyond the binge threshold. Alcohol Clin Exp Res, 2006. 30(6): p. 1006–10. 16737459 10.1111/j.1530-0277.2006.00122.x

[7] Suerken C.K., , Prevalence of marijuana use at college entry and risk factors for initiation during freshman year. Addict Behav, 2014. 39(1): p. 302–7. 24455784 10.1016/j.addbeh.2013.10.018 PMC4098711

[8] Caldeira K.M., , The occurrence of cannabis use disorders and other cannabis-related problems among first-year college students. Addict Behav, 2008. 33(3): p. 397–411. 18031940 10.1016/j.addbeh.2007.10.001 PMC2247439

[9] Wu L.T., , Alcohol use disorders and the use of treatment services among college-age young adults. Psychiatr Serv, 2007. 58(2): p. 192–200. 17287375 10.1176/appi.ps.58.2.192 PMC1831544

[10] CDC. Alcohol Use and Your Health. 2021 05/11/2021 [cited 2021 10/14/2021]; Available from: https://www.cdc.gov/alcohol/fact-sheets/alcohol-use.htm.

[11] Arria A.M., , The academic consequences of marijuana use during college. Psychol Addict Behav, 2015. 29(3): p. 564–75. 26237288 10.1037/adb0000108 PMC4586361

[12] Wood P.K., , Predicting academic problems in college from freshman alcohol involvement. J Stud Alcohol, 1997. 58(2): p. 200–10. 9065898 10.15288/jsa.1997.58.200

[13] Singleton R.A., Collegiate alcohol consumption and academic performance. J Stud Alcohol Drugs, 2007. 68(4): p. 548–55. 17568960 10.15288/jsad.2007.68.548

[14] Paschall M.J. and Freisthler B., Does heavy drinking affect academic performance in college? Findings from a prospective study of high achievers. J Stud Alcohol, 2003. 64(4): p. 515–9. 12921193 10.15288/jsa.2003.64.515

[15] Ansari W.E., Stock C., and Mills C., Is Alcohol Consumption Associated with Poor Academic Achievement in University Students? International Journal of Preventive Medicine, 2013. 4(10): p. 1175–1188. 24319558 PMC3843305

[16] Meda S.A., , Longitudinal influence of alcohol and marijuana use on academic performance in college students. PLoS One, 2017. 12(3): p. e0172213. 28273162 10.1371/journal.pone.0172213 PMC5342177

[17] Patte K.A., Qian W., and Leatherdale S.T., Marijuana and Alcohol Use as Predictors of Academic Achievement: A Longitudinal Analysis Among Youth in the COMPASS Study. J Sch Health, 2017. 87(5): p. 310–318. 28382670 10.1111/josh.12498

[18] Cronce J.M., , NIAAA’s College Alcohol Intervention Matrix. Alcohol Res, 2018. 39(1): p. 43–47. 30557147 10.35946/arcr.v39.1.07 PMC6104959

[19] Simons J.S., , Quantifying alcohol consumption: Self-report, transdermal assessment, and prediction of dependence symptoms. Addictive Behaviors, 2015. 50: p. 205–212. 26160523 10.1016/j.addbeh.2015.06.042 PMC4523087

[20] McNeely J., , Computer self-administered screening for substance use in university student health centers. Journal of American College Health, 2019. 67(6): p. 541–550. 30240331 10.1080/07448481.2018.1498852 PMC6428636

[21] Vanyukov M.M., , Measurement of the Risk for Substance Use Disorders: Phenotypic and Genetic Analysis of an Index of Common Liability. Behavior genetics, 2009. 39(3): p. 233–244. 19377872 10.1007/s10519-009-9269-9 PMC3099440

[22] Vanyukov M.M., , Liability to substance use disorders: 2. A measurement approach. Neurosci Biobehav Rev, 2003. 27(6): p. 517–26. 14599433 10.1016/j.neubiorev.2003.08.003

[23] Vanyukov M.M., , Liability to substance use disorders: 1. Common mechanisms and manifestations. Neurosci Biobehav Rev, 2003. 27(6): p. 507–15. 14599432 10.1016/j.neubiorev.2003.08.002

[24] Ridenour T.A., , Could a continuous measure of individual transmissible risk be useful in clinical assessment of substance use disorder? Findings from the National Epidemiological Survey on Alcohol and Related Conditions. Drug Alcohol Depend, 2011. 119(1–2): p. 10–7. 21715106 10.1016/j.drugalcdep.2011.05.018 PMC3184358

[25] Arria A.M., Vincent K.B., and Caldeira K.M., Measuring liability for substance use disorder among college students: implications for screening and early intervention. Am J Drug Alcohol Abuse, 2009. 35(4): p. 233–41. 20180676 10.1080/00952990903005957 PMC2829709

[26] Harris P.A., , Research electronic data capture (REDCap)--a metadata-driven methodology and workflow process for providing translational research informatics support. J Biomed Inform, 2009. 42(2): p. 377–81. 18929686 10.1016/j.jbi.2008.08.010 PMC2700030

[27] Brown A.L., , Adolescent Behavioral Characteristics Mediate Familial Effects on Alcohol Use and Problems in College-Bound Students. Subst Abuse, 2020. 14: p. 1178221820970925. 33223834 10.1177/1178221820970925 PMC7656872

[28] Brick L.A., , The intermediary role of adolescent temperamental and behavioral traits on the prospective associations between polygenic risk and cannabis use among young adults of European Ancestry. Addiction, 2021. 116(10): p. 2779–2789. 33686717 10.1111/add.15476 PMC8426427

[29] Asparouhov T. and Muthén B., Plausible values for latent variables using Mplus. Unpublished. Accessed at: http://www.statmodel.com/download/Plausible.pdf, 2010.

[30] World Health Organization, ASSIST V3.0. 2010.

[31] R Core Team, R: A language and environment for statistical computing, Computing R.F.f.S., Editor. 2014: Vienna, Austria.

[32] Carey K.B., , Individual-level interventions to reduce college student drinking: a meta-analytic review. Addict Behav, 2007. 32(11): p. 2469–94. 17590277 10.1016/j.addbeh.2007.05.004 PMC2144910

[33] Weinberger A.H., , Cannabis use among US adults with anxiety from 2008 to 2017: The role of state-level cannabis legalization. Drug Alcohol Depend, 2020. 214: p. 108163. 32707516 10.1016/j.drugalcdep.2020.108163

[34] Welsh J.W., Shentu Y., and Sarvey D.B., Substance Use Among College Students. Focus (Am Psychiatr Publ), 2019. 17(2): p. 117–127. 31975967 10.1176/appi.focus.20180037 PMC6527004

[35] Kandel D.B., Stages and pathways of drug involvement: Examining the Gateway Hypothesis. Stages and pathways of drug involvement: Examining the Gateway Hypothesis., ed. Kandel D.B.. 2002, New York, NY, US: Cambridge University Press. xvi, 384–xvi, 384.

[36] Griffin K.W. and Botvin G.J., Evidence-based interventions for preventing substance use disorders in adolescents. Child and adolescent psychiatric clinics of North America, 2010. 19(3): p. 505–526. 20682218 10.1016/j.chc.2010.03.005 PMC2916744

